# Protocol for the expression, purification, and biochemical characterization of the innate immune sensor MDA5

**DOI:** 10.1016/j.xpro.2025.104218

**Published:** 2025-11-20

**Authors:** Joe D. Joiner, Alba Herrero del Valle, Rahul Singh, Yorgo Modis

**Affiliations:** 1 Molecular Immunity Unit, Department of Medicine, University of Cambridge, MRC Laboratory of Molecular Biology, Cambridge CB2 0QH, UK; 2 Cambridge Institute of Therapeutic Immunology & Infectious Disease (CITIID), Department of Medicine, University of Cambridge, Cambridge CB2 0AW, UK; 3 Department of Pathology, University of Cambridge, Cambridge CB2 1QP, UK

**Keywords:** Biophysics, Protein Biochemistry, Protein expression and purification

## Abstract

MDA5 is one of the primary eukaryotic innate immune sensors of viruses, recognizing long double-stranded RNA (dsRNA). Here, we present procedures for the recombinant expression and purification of murine MDA5 from *E. coli*. We describe the steps to purify MDA5 in high yields for downstream experiments and procedures to determine the ATPase activity and RNA-binding properties of purified MDA5. These approaches can be used to produce disease-associated mutants of MDA5, to uncover the biochemical mechanisms underpinning known disease phenotypes.

For complete details on the use and execution of this protocol, please refer to Singh et al.^1^

## Highlights

• Procedures for the expression and purification of recombinant mouse MDA5 from *E. coli*
• Procedures for quantifying the ATPase and RNA-binding activities of purified MDA5
• Visualization and biophysical characterization of MDA5-dsRNA filaments

## Abstract (editor highlights)

Publisher’s note: Undertaking any experimental protocol requires adherence to local institutional guidelines for laboratory safety and ethics.

## Graphical abstract

**Figure:**
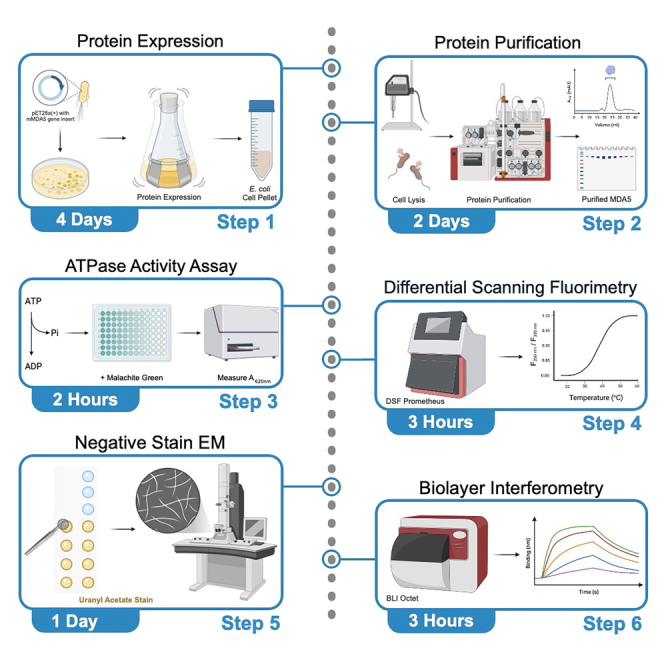

## Before you begin

Melanoma Differentiation Associated protein 5 (MDA5) is a retinoic acid-inducible gene I (RIG-I)-like helicase and one of the main innate immune sensors of cytosolic double-stranded RNA (dsRNA), recognizing dsRNAs longer than 100 base pairs (bp) in length. The *IFIH1* gene, encoding MDA5, is a hotspot for natural variants with varied clinical associations, including both increased susceptibility to, and protection against, autoinflammatory disorders. Different mutations in the *IFIH1* gene cause disease-related phenotypes through a range of underlying molecular mechanisms.^1^^,^^2^^,^^3^

This protocol outlines the expression and purification of recombinant mouse MDA5 (mMDA5), which has higher solubility and higher expression yields compared to the homologous human protein, allowing for expression of MDA5 in *Escherichia coli* (*E. coli*) without the need for additional fusion protein tags. In the construct used in this protocol (mMDA5Δ646-663), residues 646–663, which are situated in the flexible L2 surface loop of the helicase 2 insert (Hel2i) domain, are deleted to improve protein solubility without affecting the dsRNA binding, ATPase or interferon signaling activities of MDA5.^4^^,^^5^

This protocol can be used to purify a variety of MDA5 mutants, including both gain- and loss-of-function variants, and to study the biochemical properties underpinning their associated disease phenotypes. The purified protein can be used for *in vitro* structural and biochemical characterization, and this protocol additionally includes instructions for methods used to study the ATPase activity, RNA-binding, and filament-forming properties of purified MDA5 proteins. To investigate the biochemical properties of purified MDA5, RNA can be transcribed *in vitro*. RNA synthesis can be performed in advance of protein production and stored at −80°C for future use.

### Innovation

This protocol combines existing methods for the expression and purification of mMDA5 into a single, updated protocol. The optimized construct encoding mMDA5 allows for protein expression in *E. coli*, providing a faster, more straightforward protocol than using alternative eukaryotic expression systems. This protocol for MDA5 expression does not require the presence of additional fusion tags, which would result in a more time-consuming purification protocol involving the cleavage and removal of the fusion protein. The described biochemical assays provide updated methods for the fast validation of protein quality following purification, with optimized conditions for studying MDA5 activity.

### RNA synthesis

**Timing: 2 days**

1. Amplification of the DNA template.
  a. Amplify the DNA template of desired length by PCR.
  b. Purify DNA templates using a PCR purification or gel extraction kit.

**CRITICAL:** The T7 promoter sequence (5′ TAATACGACTCACTATAG 3′) should be added at the 5′ end of the primer used to amplify the coding strand, to allow for downstream transcription (Step 2).

***Note:*** The T7 promoter sequence can also be added to both primers used to amplify the template in this step. This will result in co-transcription of both RNA strands in step 2, yielding a dsRNA duplex from a single *in vitro* transcription reaction.

**Pause point:** DNA templates can be stored at −20°C for long-term storage and used for subsequent *in vitro* transcription reactions at a later date.

2. Perform *in vitro* transcription of desired RNA strands using the MEGAscript T7 Transcription Kit (Invitrogen, cat. no. AM1333) or HiScribe T7 High Yield RNA Synthesis Kit (New England BioLabs, cat. no. E2040S), according to the manufacturer’s instructions.
3. DNase digestion to remove the DNA template.
  a. Add 1–2 μL of TURBO DNase to each *in vitro* transcription reaction.
  b. Incubate at 37°C for 15–30 minutes.
4. Purify the synthesized RNA using the Monarch RNA Cleanup Kit (500 μg) (New England BioLabs, cat. no. T2050L), following the manufacturer’s instructions.

***Note:*** Synthesized RNA was eluted using Nuclease-Free Duplex Buffer (Integrated DNA Technologies) instead of nuclease-free water, as recommended for the storage of duplexed RNA.

5. Check purity of synthesized RNA by denaturing gel electrophoresis: Run a sample of the synthesized RNA on a TBE-urea polyacrylamide gel (<300 bp) or denaturing (formaldehyde) agarose gel to confirm synthesized RNA is of the correct size and to assess purity and integrity of samples.

***Optional:*** In case there are multiple RNA bands (e.g., Due to premature transcription termination or run off by-products), the RNA can be further purified by TBE-urea-PAGE or agarose gel electrophoresis and gel extraction; however, note that this may damage the RNA.

6. Anneal complementary RNA strands together.
  a. Mix the complementary RNA strands together, at equal final concentrations.
  b. Heat RNA samples to 95°C and incubate for 5 min to eliminate secondary structure.
  c. Cool slowly to room temperature (∼22°C) over 2 h to enable annealing of complementary strands of RNA.
7. Store the purified RNA at −80°C.

***Note:*** Purified RNA can be stored at −80°C for at least one year. Integrity of the synthesized product and older RNA stocks should be verified by denaturing agarose gel electrophoresis/ urea-PAGE before use.

***Note:*** For downstream BLI experiments, biotinylation of the 3′-end of RNA is required. RNA biotinylation can be achieved using the Pierce RNA 3’ End Biotinylation Kit (Thermo Scientific, cat. no. 20160) and following the manufacturer’s instructions.

### Sample preparation

**Timing: 1–2 days**

8. Cloning of the mMDA5 gene into the pET28a(+) plasmid.

***Note:*** The gene encoding mMDA5Δ646-663 is cloned into the pET28a(+) (Novagen) expression vector, in frame with an N-terminal His_6_-tag and a thrombin cleavage site for expression in *E. coli* and subsequent purification by Ni affinity chromatography. The amino acid sequence of the translated protein is:

MGSSHHHHHHSSGLVPRGSHMSIVCSAEDSFRNLILFFRPRLKMYIQVEPVLDHLIFLSAETKEQILKKINTCGNTSAAELLLSTLEQGQWPLGWTQMFVEALEHSGNPLAARYVKPTLTDLPSPSSETAHDECLHLLTLLQPTLVDKLLINDVLDTCFEKGLLTVEDRNRISAAGNSGNESGVRELLRRIVQKENWFSTFLDVLRQTGNDALFQELTGGGCPEDNTDLANSSHRDGPAANECLLPAVDESSLETEAWNVDDILPEASCTDSSVTTESDTSLAEGSVSCFDESLGHNSNMGRDSGTMGSDSDESVIQTKRVSPEPELQLRPYQMEVAQPALDGKNIIICLPTGSGKTRVAVYITKDHLDKKKQASESGKVIVLVNKVMLAEQLFRKEFNPYLKKWYRIIGLSGDTQLKISFPEVVKSYDVIISTAQILENSLLNLESGDDDGVQLSDFSLIIIDECHHTNKEAVYNNIMRRYLKQKLRNNDLKKQNKPAIPLPQILGLTASPGVGAAKKQSEAEKHILNICANLDAFTIKTVKENLGQLKHQIKEPCKKFVIADDTRENPFKEKLLEIMASIQTYCQKSPMSDFGTQHYEQWAIQMEKKAAKDGNRKDRVCAEHLRKYNEALQINDTIRMIDAYSHLETFYTDEKEKKFAVLNNDSKKSLKLDETDEFLMNLFFDNKKMLKKLAENPKYENEKLIKLRNTILEQFTRSEESSRGIIFTKTRQSTYALSQWIMENAKFAEVGVKAHHLIGAGHSSEVKPMTQTEQKEVISKFRTGEINLLIATTVAEEGLDIKECNIVIRYGLVTNEIAMVQARGRARADESTYVLVTSSGSGVTEREIVNDFREKMMYKAINRVQNMKPEEYAHKILELQVQSILEKKMKVKRSIAKQYNDNPSLITLLCKNCSMLVCSGENIHVIEKMHHVNMTPEFKGLYIVRENKALQKKFADYQTNGEIICKCGQAWGTMMVHKGLDLPCLKIRNFVVNFKNNSPKKQYKKWVELPIRFPDLDYSEYCLYSDED.

9. Buffer preparation.

***Note:*** All buffers for purification can be prepared in advance and stored at 4°C, as described in the materials and equipment section.

## Key resources table

**Table:** 

REAGENT or RESOURCE	SOURCE	IDENTIFIER
**Bacterial and virus strains**		

*Escherichia coli* Rosetta 2(DE3)pLysS competent cells	Novagen	Cat#71403

**Chemicals, peptides, and recombinant proteins**		

Kanamycin sulfate	SERVA	Cat#26899
Chloramphenicol	Thermo Scientific	Cat#B20841
Tryptone	Merck Millipore	Cat#T9410
Yeast extract	Sigma-Aldrich	Cat#Y1625
Isopropyl-beta-D-thiogalactoside (IPTG)	Protein Ark	Cat#GEN-S-02122
cOmplete EDTA-free protease inhibitor cocktail	Roche	Cat#11873580001
Salt active nuclease	Merck	Cat#SRE0015
TURBO DNase	Invitrogen	Cat#AM2238
Recombinant mouse MDA5	This study	N/A
Bovine serum albumin (BSA)	Sigma-Aldrich	Cat#A4503
HEPES	Sigma-Aldrich	Cat#H3375
Sodium chloride (NaCl)	Sigma-Aldrich	Cat#S9888
Magnesium chloride (MgCl_2_)	Sigma-Aldrich	Cat#M9272
Magnesium sulfate (MgSO_4_)	Sigma-Aldrich	Cat#M1880
Potassium chloride (KCl)	Sigma-Aldrich	Cat#P3911
Glycerol	Sigma-Aldrich	Cat#G7893
Tris(2-carboxyethyl)phosphine (TCEP)	Merck	Cat#646547
Imidazole	Sigma-Aldrich	Cat#56750
Dithiothreitol (DTT)	Thermo Scientific	Cat#R0862
Adenosine 5′-triphosphate (ATP) disodium salt hydrate	Sigma-Aldrich	Cat#A7699

**Critical commercial assays**		

QIAquick PCR purification kit	QIAGEN	Cat#28104
QIAquick gel extraction kit	QIAGEN	Cat#28704
HiScribe T7 high yield RNA synthesis kit	New England Biolabs	Cat#E2040S
MEGAscript T7 transcription kit	Invitrogen	Cat#AM1333
Monarch RNA cleanup kit	New England Biolabs	Cat#T2050L
Pierce RNA 3′ end biotinylation kit	Thermo Scientific	Cat#20160
ATPase/GTPase activity assay kit	Sigma-Aldrich	Cat#MAK113

**Recombinant DNA**		

pET28a(+)	Novagen	Cat#69864
pET28a(+)-mMDA5Δ646-663	This study	N/A

**Software and algorithms**		

PR.ThermControl v.2.3	NanoTemper	nanotemper.com
PR.Stability Analysis v.1.1	NanoTemper	nanotemper.com
Fiji v.2.16	Schindelin et al.^6^	https://fiji.sc/
Octet Data Acquisition software v.11.1	Sartorius	sartorius.com
Octet Data Analysis HT software v.11.1	Sartorius	sartorius.com
Prism v.10	GraphPad	graphpad.com

**Other**		

Äkta protein purification system	Cytiva	N/A
HisTrap HP His tag purification column	Cytiva	Cat#17524801
RESOURCE Q anion exchange chromatography column	Cytiva	Cat#17117701
Superdex 200 Increase 10/300 GL size-exclusion column	Cytiva	Cat# 28990944
NanoDrop spectrophotometer	N/A	N/A
Ultrasonic processor VCX 500	Sonics & Materials, Inc	Cat#VCX500
Ultrasonication standard probe	Sonics & Materials, Inc.	Cat#630-0220
Amicon Ultra centrifugal filters, 50 kDa MWCO	Merck Millipore	Cat#UFC9050
Econo-Pac 10DG desalting columns	Bio-Rad	Cat#7322010
Syringe filter, 0.45 μm	Fisherbrand	Cat#15191499
Syringe filter, 0.2 μm	Fisherbrand	Cat#15181499
Syringe filter, 0.1 μm	Sartorius	Cat#16553-K
Falcon 96-well clear flat bottom plates	Corning	Cat#351172
CLARIOstar Plus microplate reader	BMG Labtech	N/A
Prometheus instrument	NanoTemper	N/A
Prometheus NT.48 Series nanoDSF grade standard capillaries	NanoTemper	Cat#PR-C002
Carbon films on copper grids (300 mesh)	Agar Scientific	Cat#AGS160-3
Formvar carbon films on copper grids (300 mesh)	Agar Scientific	Cat#AGS162-3
120 kV Tecnai G2 Spirit transmission electron microscope	FEI/Thermo Scientific	N/A
Octet biolayer interferometry system	Sartorius	N/A
Octet streptavidin (SA) biosensors	Sartorius	Cat#18-5019
96-well microplate, black	Greiner Bio-One	Cat# 655209

## Materials and equipment

***Alternatives:*** This protocol uses an ÄKTA chromatography system (Cytiva) for protein purification. Several different ÄKTA systems are available that can be used for this protocol, along with other FPLC systems from other manufacturers. A sonication device is used for cell lysis prior to purification. Sonicator models from different suppliers or alternative methods for cell lysis, such as using a French press, can all be used for the cell lysis step.

***Alternatives:*** In this protocol, a NanoDrop spectrophotometer is used to measure protein concentrations for assays. Spectrophotometers from different manufacturers can all be used, along with alternative methods for measuring protein concentrations, such as cuvette-based spectrophotometers.

***Alternatives:*** A CLARIOstar Plus microplate reader (BMG Labtech) was used to measure absorbance in the ATPase assay. Alternative microplate readers can be used, provided they can measure absorbance at 620 nm. Prometheus (NanoTemper) and Octet (Sartorius) instruments were used for nanoDSF and BLI measurements, respectively. Both manufacturers supply multiple versions of each of these instruments, all of which can be used in the protocols described here. A 120 kV Technai G2 Spirit transmission electron microscope (TEM) was used for imaging negative stain grids. Other high resolution TEMs (such as the Technai F20) can also be used for collecting micrographs.

**Table:** 2xTY Media

Reagent	Final concentration	Amount
Tryptone	16 g L^−1^	16 g
Yeast extract	10 g L^−1^	10 g
NaCl	5 g L^−1^	5 g
ddH_2_O	N/A	to 1 L
**Total**	**N/A**	**1 L**

Adjust pH to 7.4 before making up final volume to 1 L. Autoclave buffer at 121°C for 15 mins. Add kanamycin and chloramphenicol to final concentrations of 50 μg mL^−1^ and 30 μg mL^−1^, respectively. Autoclaved media can be stored at room temperature (20°C–25°C) for up to 1 week.

**Table:** Lysis Buffer

Reagent	Final concentration	Amount
HEPES pH 7.7 (1 M)	30 mM	30 mL
NaCl (5 M)	500 mM	100 mL
MgCl_2_ (1 M)	5 mM	5 mL
Glycerol (20%)	5% (v/v)	250 mL
TCEP (0.5 M)	1 mM	2 mL
ddH_2_O	N/A	to 1L
**Total**	**N/A**	**1 L**

Filter (0.22 μm filter) and store at 4°C for up to 3 months.

**Table:** Wash Buffer

Reagent	Final concentration	Amount
HEPES pH 7.7 (1 M)	30 mM	30 mL
NaCl (5 M)	500 mM	100 mL
Imidazole (1 M)	20 mM	20 mL
Glycerol (20%)	5% (v/v)	250 mL
TCEP (0.5 M)	1 mM	2 mL
ddH_2_O	N/A	to 1L
**Total**	**N/A**	**1 L**

Filter (0.22 μm filter) and store, protected from light, at 4°C for up to 3 months.

***Alternatives:*** Wash buffer for the Ni affinity step can also be constituted by mixing lysis and elution buffers at a ratio of 90:10.

**Table:** Elution Buffer

Reagent	Final concentration	Amount
HEPES pH 7.7 (1 M)	30 mM	30 mL
NaCl (5 M)	300 mM	60 mL
Imidazole (1 M)	250 mM	250 mL
Glycerol (20%)	5% (v/v)	250 mL
TCEP (0.5 M)	1 mM	2 mL
ddH_2_O	N/A	to 1L
**Total**	**N/A**	**1 L**

Filter (0.22 μm filter) and store, protected from light, at 4°C for up to 3 months.

***Alternatives****:* The buffer can be prepared without imidazole if being prepared in advance. Add imidazole, adjust the pH, and filter buffer directly before use.

**Table:** Buffer A

Reagent	Final concentration	Amount
HEPES pH 7.7 (1 M)	20 mM	20 mL
NaCl (5 M)	50 mM	10 mL
DTT (1 M)	1 mM	1 mL
ddH_2_O	N/A	to 1L
**Total**	**N/A**	**1 L**

Prepare without DTT, filter (0.22 μm filter) and store at 4°C for up to 3 months. Add DTT and degas buffer directly before use.

**Table:** Buffer B

Reagent	Final concentration	Amount
HEPES pH 7.7 (1 M)	20 mM	20 mL
NaCl (5 M)	1 M	200 mL
DTT (1 M)	1 mM	1 mL
ddH_2_O	N/A	to 1L
**Total**	**N/A**	**1 L**

Prepare without DTT, filter (0.22 μm filter) and store at 4°C for up to 3 months. Add DTT and degas buffer directly before use.

**Table:** Size Exclusion Buffer

Reagent	Final concentration	Amount
HEPES pH 7.7 (1 M)	20 mM	20 mL
KCl (3 M)	150 mM	50 mL
DTT (1 M)	1 mM	1 mL
ddH_2_O	N/A	to 1L
**Total**	**N/A**	**1 L**

Prepare without DTT, filter (0.22 μm filter) and store at 4°C for up to 3 months. Add DTT and degas buffer directly before use.

**Table:** ATPase Assay Buffer

Reagent	Final concentration	Amount
HEPES pH 7.7 (1 M)	20 mM	200 μL
KCl (3 M)	150 mM	500 μL
MgSO_4_ (1M)	4 mM	40 μL
DTT (1 M)	1 mM	10 μL
ddH_2_O	N/A	to 10 mL
**Total**	**N/A**	**10 mL**

Prepare without DTT, filter (0.22 μm filter) and store at 4°C for up to 3 months. Add DTT directly before use.

***Alternatives:*** 40 μL of MgSO_4_ (1 M) can be added directly to Size Exclusion Buffer (10 mL), and the assay buffer re-filtered before use.

**Table:** BLI Assay Buffer

Reagent	Final concentration	Amount
HEPES pH 7.7 (1 M)	20 mM	1 mL
KCl (3 M)	150 mM	2.5 mL
DTT (1 M)	1 mM	50 μL
BSA	2 mg mL^−1^	100 mg
ddH_2_O	N/A	to 50 mL
**Total**	**N/A**	**50 mL**

Prepare buffer, filter (0.10 μm filter) and degas directly before use. Store at 4°C for up to 1 week.

***Alternatives:*** BSA can be solubilized directly in Size Exclusion Buffer, and the assay buffer re-filtered and degassed immediately before use.

## Step-by-step method details

### Expression of mouse MDA5

**Timing: 4 days**

This section describes the large-scale expression of recombinant mouse MDA5 in *E. coli*. The collected cell pellet can be stored for downstream purification.

***Note:*** Expression is carried out in flasks, and the total volume of cell culture can be scaled according to the needs of the end-user, typically ranging from 1.6 to 5 L (6 × 800 mL cultures). We recommend diluting the overnight starter culture 1:50–1:100 for large-scale growth, and the amount of the starter culture should be adjusted to ensure a sufficient volume is available for inoculation.

***Note:*** The mMDA5Δ646-663 construct contains an N-terminal His_6_-tag, followed by a thrombin cleavage site, for affinity purification. Removal of the tag is not required in the purification procedure described in this protocol, and the presence of the tag does not affect the protein ATPase activity, RNA binding or filament formation, as determined in the steps outlined in this protocol.

1. Transformation of *E. coli* Rosetta 2(DE3)pLysS competent cells (Novagen Cat#71403) with the pET28a(+) expression vector containing the gene encoding mMDA5Δ646-663.

***Note:*** LB agar plates should be supplemented with both chloramphenicol (Rosetta pLysSRARE2 plasmid resistance) and kanamycin (pET28a(+) plasmid resistance).

2. Inoculation of starter culture.
  a. Inoculate an overnight starter culture of 50 mL 2xTY media (with 30 μg mL^−1^ chloramphenicol and 50 μg mL^−1^ kanamycin) with a single colony from the LB agar plate.
    ***Note:*** Volume of overnight cultures can be scaled up, in accordance with the flasks used for large scale expression (step 3).
  b. Incubate overnight at 37°C with shaking (170 rpm).
3. Large scale overexpression.
  a. Inoculate 800 mL of 2xTY media (supplemented with 30 μg mL^−1^ chloramphenicol and 50 μg mL^−1^ kanamycin) with 8–16 mL of overnight culture (1:50–1:100 dilution).
    ***Note:*** Use of baffled flasks may aid bacterial growth and reduce the time needed to reach the required optical density (OD_600_) for induction.
  b. Incubate cells at 37°C (170 rpm) until an OD_600_ of 0.6–0.8 (∼2.5–3 hours).
    i. Incubate cells at 37°C until OD_600_ 0.4–0.6.
    ii. Reduce temperature of incubator to 16°C (170 rpm) once an OD_600_ of 0.4 is reached. Final OD_600_ at point of induction should be in the range of 0.6–0.8.
  c. Induce cells by adding 400 μL of 1 M isopropyl-β-D-1-thiogalactopyranoside (IPTG) (final IPTG concentration of 0.5 mM).
  d. Incubate cells at 16°C overnight with shaking (150 rpm).
  e. Collect cells by centrifugation (∼8000 × *g*) for 10–15 min at 4°C. Discard the supernatant.
  f. Wash the pellet.
    i. Resuspend in 30–50 mL ice-cold 1× PBS.
    ii. Centrifuge at 4°C (∼4000 × *g*) until cells are fully pelleted (supernatant is clear). Discard supernatant.
      ***Note:*** Exact centrifuge time will depend on the pellet size, and may take up to 30 min.
    iii. Flash-freeze the pellet in liquid N_2_.
  g. Store cell pellet at −80°C or proceed directly to protein purification.
    ***Note:*** The cell pellet obtained by following this protocol typically weighs between 6–12 g/L of *E. coli* culture.
    **Pause point:** Cell pellet can be stored at −80^o^C for up to 1 year.
    ***Note:*** The final yield of purified protein may be improved when proceeding directly to protein purification or short-term storage at −80°C, compared to longer-term storage greater than one month.

### Purification of mouse MDA5

**Timing: 2 days**

This section describes the purification of mouse MDA5 from the soluble fraction of the *E. coli* cell pellet. A three-step purification protocol is used, consisting of immobilized metal affinity chromatography (IMAC), anion exchange chromatography and size exclusion chromatography (SEC), resulting in a high final purity of MDA5.

**Figure 1:**
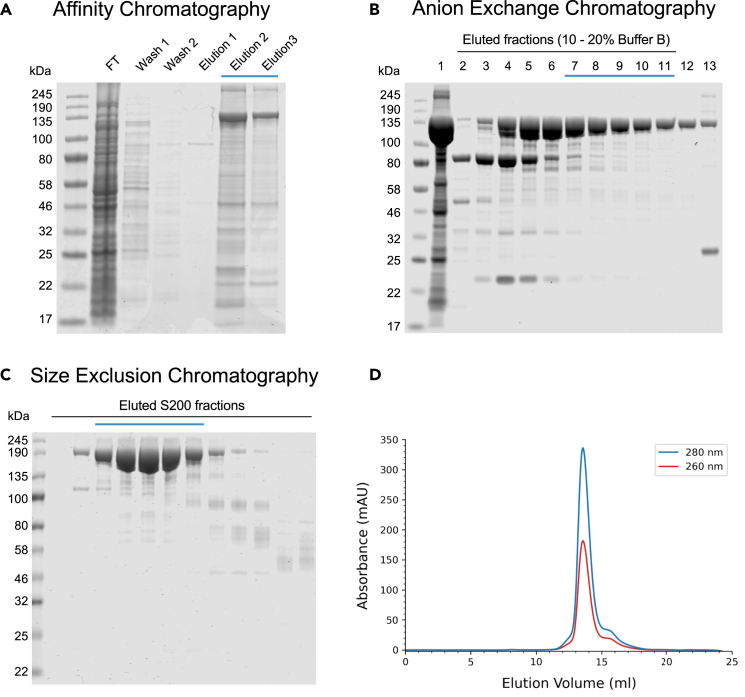
Purification of mMDA5 (A–C) Coomassie-stained SDS gels for fractions eluted for each step of the MDA5 purification protocol: (A) Ni affinity chromatography, (B) anion exchange chromatography, (C) size exclusion chromatography (SEC). Samples were resolved on 4%–12% Bis-Tris gels and stained with Coomassie. Fractions pooled and used for subsequent purification steps, or biochemical assays, are underlined in blue. Anion exchange chromatography samples were taken from fractions corresponding to the peaks eluted at each step of the protocol: 1) flow through; 2–11) elution step 2 (10%–20% Buffer B); 12) elution step 3 (20%–40% Buffer B); 13) elution step 4 (40%–100% Buffer B). FT: Flow through. (D) Elution trace for the SEC step of MDA5 purification. MDA5 was purified using a Superdex200 Increase 10/300 GL size-exclusion chromatography column, and absorbance monitored at 260 and 280 nm.

4. Cell lysis.
  a. Thaw cell pellet and resuspend in lysis buffer (30 mL lysis buffer per 800mL of initial cell culture/5 mL lysis buffer per g of harvested cell pellet).
  b. Add one EDTA-free Protease Inhibitor Cocktail tablet and Salt Active Nuclease (10 μL per 50 mL of sample) to the resuspended pellet.
  c. Sonicate lysate for 5–8 min (program: 5 seconds on, 10 seconds off, 200-watt (40%) power), maintaining cell suspension on ice throughout.
    ***Note:*** A 500-watt ultrasonic processor with 1/2″ diameter probe was used in the protocol described here. The protocol for sonication can be adapted, depending on the power output of the sonication device available, to ensure 200-watt of energy is delivered during this step. The probe size should be selected based on the cell lysate volume – a 1/2″ (12 mm) diameter probe is recommended for 50–250 mL sample volumes.
    ***Alternatives:*** Other methods for bacterial cell lysis (e.g., French press) can also be used instead of sonication.
  d. Centrifuge cell lysate at 38,000 × *g* for 1 hour at 4°C.
  e. Filter supernatant through a 0.45 μm filter. Discard pellet.
5. Affinity chromatography.
  a. Equilibrate HisTrap column with > 3 column volumes (CV) of lysis buffer.
  b. Load the filtered supernatant to the column, collecting the flow through.
  c. Wash the column with at least 15–20 CV of wash buffer.
    ***Note:*** If using an Äkta for this step, wait until Abs_260nm_ and Abs_280nm_ have returned to the baseline level. If using a peristatic pump or gravity column, measure Abs_260nm_ and Abs_280nm_ of column eluate. Once the absorbance values of the column flow through are zero (relative to the wash buffer), proceed to sample elution.
  d. Elute the protein by washing the column with 5–10 CV of elution buffer, collecting the eluate in fractions (typically 1 CV in volume each).
  e. Determine which fractions contain purified MDA5 by SDS-PAGE (Figure 1A).
    ***Note:*** A gravity column with Ni-NTA agarose resin/ magnetic beads can be used instead of a prepacked HisTrap column for this purification step. In this instance, then 3–5 mL of Ni slurry (50%) should be used depending on the amount of lysate, and the volumes of buffers adjusted accordingly. Binding capacities of the resin depends on the exact column/ agarose used but are typically 20–40 mg His-tagged protein per mL of Ni resin.
    ***Note:*** If using a prepacked HisTrap column, a flow rate of 1–2 mL min^−1^ is recommended for this step.
6. Anion exchange chromatography.
  a. Concentrate eluted protein.
    i. Combine eluted fractions containing MDA5.
    ii. Equilibrate Amicon Ultra Centrifugal Filters (50 kDa MWCO) with lysis buffer.
    iii. Concentrate protein sample until volume is ≤ 3 mL.
  b. Desalt the protein sample using a desalting column.
    i. Equilibrate the desalting column with 20 mL of buffer A.
    ii. Load protein sample (max 3 mL). Allow sample to completely enter the sample and discard flow through.
    iii. Add 4 mL of buffer A to the column and collect the eluate (containing desalted MDA5).
      ***Note:*** Sample loading and eluate volumes refer to use of an Econo-Pac 10DG desalting column (Bio-Rad) and may need to be adjusted slightly depending on the exact desalting column used, according to the manufacturer’s instructions.
  c. Run Anion Exchange column.
    i. Equilibrate the Resource Q column with 10 CV of Buffer A.
    ii. Load the desalted MDA5 sample (4 mL) onto the column, and wash the column with 5 CV of Buffer A.
    iii. Elute the protein by washing the column with an increasing gradient of Buffer B, and a flow rate of 2 mL min^−1^. A gradient elution is performed stepwise, with the following final percentage of Buffer B reached over the stated elution volume: 10% Buffer B (15 CV), 20% B (60 CV), 40% (10 CV), 100% (10 CV). MDA5 (free from RNA contamination) should elute during the second step (at 10%–15% Buffer B, Figure 1B).
      ***Note:*** If a suitable wavelength detector is available, UV absorbance can be monitored at 260 nm in addition to 280 nm, to check for nucleic acid contamination in the eluted fractions.
      **Pause point:** Protein can be stored overnight at 4°C after elution of the Resource Q column.
      ***Note:*** Alternative strong anion exchange columns (other than the Resource Q column used here) can also be used to perform this purification step.
7. Size exclusion chromatography.
  a. Equilibrate the Superdex 200 Increase 10/300 GL size-exclusion column with 1–2 CV of SEC buffer.
  b. Concentrate fractions containing MDA5 (as determined by SDS-PAGE).
    i. Equilibrate Amicon Ultra Centrifugal Filters (50 kDa MWCO) with SEC buffer.
    ii. Concentrate MDA5 to final volume ≤ 500 μL.
  c. Load concentrated protein sample to the Superdex 200 size-exclusion column and elute the purified MDA5 with a flow rate of 0.75 mL min^−1^ (Figure 1D).

***Note:*** UV absorbance should be monitored at 280 nm to determine which fractions contain the eluted MDA5 and, optionally, also at 260 nm to check for nucleic acid contamination.

***Note:*** For larger-scale protein preparations, a Superdex 200 16/60 size column can be used, and the loading volume increased up to 3 mL.

8. Storage of protein samples.

Pool fractions containing pure MDA5, as determined by SDS-PAGE (Figure 1C).

***Note:*** Purified MDA5 can be used immediately for biochemical assays and stored at 4°C for 1–2 days.

***Note:*** For longer-term storage, glycerol should be added to the final MDA5 sample (to a final concentration of 5%), and aliquots flash frozen in liquid nitrogen and stored at 80°C for future experiments.

**Pause point:** Purified MDA5 can be stored at −80°C for at least one year.

***Note:*** If using frozen aliquots of MDA5 for future assays, the thawed aliquots should be rerun through a size exclusion or desalting column in SEC buffer, to verify protein integrity and remove glycerol before performing biochemical assays. Avoid multiple freeze-thaw cycles.

***Note:*** If issues with protein aggregation and precipitation are observed upon thawing of aliquots, the concentration of glycerol used for storage can be increased up to 10%–20% (see troubleshooting).

***Note:*** For ATPase assays, freshly purified MDA5 should be used, to avoid possible loss of ATPase activity observed after freezing. We did not observe any change in RNA binding affinity of MDA5 after one freeze-thaw cycle.

### ATPase activity assay

**Timing: 1–1.5 h**

This assay is used to determine the ATPase activity of purified MDA5 using the ATPase/GTPase Activity Assay Kit (Sigma-Aldrich, cat. no. MAK113), and this protocol is adapted from the manufacturer’s instructions. The ATPase activity of purified MDA5, in the presence of different RNAs, is determined by measuring the amount of free phosphate present in the solution as a product of ATP hydrolysis, using a standard curve. Reactions are carried out in clear, flat-bottom 96-well plates.

**CRITICAL:** For the ATPase assay, freshly purified MDA5 should be used and the protein not freeze-thawed. ATP should be prepared fresh, or flash frozen in liquid N_2_, stored at −20°C, and used within 1 month. Avoid repeat freeze-thaw cycles.

***Note:*** All samples should be run in triplicate and all standards and controls run in at least duplicate. The mean value of technical replicates within the same plate is used as one experimental replicate.

***Note:*** If determining the phosphate generated at specific time points, reactions can be quenched by the addition of EDTA (at a final concentration of 20 mM) at the desired time point, before the addition of, and incubation with, malachite green.

9. Prepare phosphate standard solutions.
  a. Prepare a 50 μM Phosphate Standard by mixing 25 μL of the 1 mM Phosphate Standard with 475 μL of nuclease-free water.
  b. Prepare standard dilutions by mixing the 50 μM phosphate standard with nuclease-free water to give standards of the following concentrations: 0, 12.5, 25, 37.5, 50 μM.
  c. Transfer 40 μL of each standard to individual wells of the 96-well plate.
10. Sample preparation.
  a. Prepare, in triplicate, 10 μL of each assay sample, containing 180 nM (20.9 μg mL^−1^) MDA5, 25 ng (2.5 ng μL^−1^) RNA in assay buffer (see materials and equipment section). Transfer into individual wells of the plate.
  b. A negative control (NEC) is prepared by adding 10 μL of assay buffer into duplicate wells of the plate. An additional protein-only negative control (containing 180 nM MDA5 in 10 μL assay buffer) should also be prepared, to confirm lack of ATP hydrolysis in the absence of RNA.
  c. A buffer-only control, to check for possible phosphate present in the protein sample buffer, is prepared by adding 40 μL of assay buffer, to duplicate wells of the plate.
  d. Prepare a 4 mM stock of ATP (in nuclease-free water).
  e. Prepare the reaction mixture, by mixing 10 μL of the 4 mM ATP stock with 20 μL of the assay buffer. 30 μL of reaction mixture is needed for each sample.
11. Perform ATPase assay.
  a. Add 30 μL of the reaction mixture to each of the assay samples and negative control wells (but not to the phosphate standards or buffer-only control sample wells).
  b. Incubate all samples at 37°C for 15 min.
  c. Quench the reaction by adding 200 μL of the malachite green reagent to each sample, control, and phosphate standard well, and incubate at room temperature (∼22°C) for 30 min.

***Note:*** It is recommended to use a multichannel pipette for this step.

12. Measure the absorbance at 620 nM using a plate reader.
13. Data analysis.
  a. Create a standard curve by plotting the concentration of the phosphate standards against the corresponding ΔOD_620nm_ values (OD_620nm_ - OD_620nm_(0 μM)).
  b. Calculate ΔOD values for assay samples by subtracting the average ΔOD of the NEC samples from each of the assay sample OD values.
  c. Calculate the phosphate generated in each sample by extrapolation from the standard curve.

### Nanoscale differential scanning fluorimetry

**Timing: 2–3 h**

This assay is used to determine the thermal stability of purified proteins, and whether different mutations in MDA5 impact protein stability, using the Prometheus instrument (NanoTemper). The increased thermal stability of MDA5-RNA filaments, compared to the apo protein, means that this assay can also be used to quickly determine whether the purified protein can bind to, and form stable filaments on, long RNAs, and compare the stability of MDA5 filaments formed on different RNA molecules.

14. Sample preparation.
  a. Prepare samples containing 1 μM MDA5 and, if required, 15 ng μL^−1^ 1-kb dsRNA in size exclusion buffer, in a volume of 20 μL.
  b. For MDA5-RNA complex samples, incubate samples at room temperature (∼22°C) for 30 min.

***Note:*** For protein-only samples (without RNA present), this extended incubation step is not required prior to loading of capillaries.

**Figure 2:**
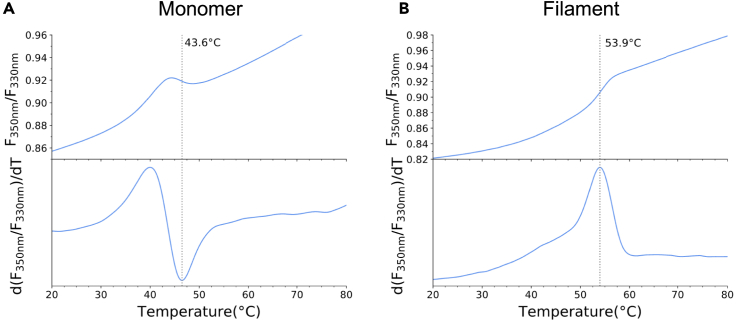
Thermal stability of purified MDA5, as determined by nanoDSF Thermal unfolding plots of (A) MDA5 (1 μM) and (B) MDA5 filaments (constituted from the incubation of 1 μM MDA5 with 15 ng μL^−1^ 1-kb dsRNA). Intrinsic protein fluorescence at 330 and 350 nm was measured, and the fluorescence ratio (F_350_/F_330_) plotted as a function of temperature. Plotted curves are the average taken from at least 5 replicate samples. Lower plot shows the first derivative of the fluorescence ratio (d(F_350_/F_330_)/dT), to visualize the infliction point (corresponding to the melting temperature) in the upper plot. Gray lines indicate the calculated melting temperatures.

15. Load samples to Prometheus NT.48 standard capillaries (NanoTemper).
16. Perform DSF run using PR.ThermControl software (NanoTemper).
  Use a temperature gradient of 1°C min^−1^ over the temperature range of 20°C–80°C.
17. Analyze data using PR.StabilityAnalysis software (NanoTemper).
  a. Merge data from individual replicates and exclude outliers from the analysis.
  b. Create a region of interest covering the unfolding transition (e.g., 40 to 70°C) and calculate melting temperatures using a two-state fit (Figure 2).

### Negative stain electron microscopy

**Timing: 3 h**

Negative stain electron microscopy can be used to easily visualize whether purified MDA5 can form filaments on RNA. Analysis of obtained images can be used to quantify the lengths of MDA5 filaments and compare the filament-forming properties of different MDA5 mutants and RNA ligands.

***Alternatives:*** In this protocol, uranyl acetate (2% solution) is used for negative staining. Other radioactive (such as uranyl formate) or non-radioactive (such as Uranyless) staining reagents can also be used instead.

***Alternatives:*** Grids with either carbon or formvar/carbon films on 200–400 mesh copper can be used for negative staining. Formvar/carbon composite films have increased strength and stability compared to carbon films, allowing for easier handling, although the resulting images may have a higher background/ reduced contrast compared to grids with carbon films.

18. Sample preparation.
  a. Prepare samples containing 200 nM MDA5 and 3 ng μL^−1^ 1-kb dsRNA in size exclusion buffer.
  b. Incubate samples at room temperature (∼22°C) for 30 min.
19. Prepare negative stain grids.
  a. Glow discharge grids at 25 mA for 60 s.
  b. Pipette out 3 × 10 μL drops of MilliQ water and 4 × 10 μL drops of 2% uranyl acetate in a row on a piece of parafilm, for each grid being prepared.
  c. Apply 3–4 μL of sample directly onto the grid and incubate for 2 minutes.
  d. Blot by touching the edge of the grid on a filter paper.
  e. Wash by picking up one drop of water and then blotting edge on the filter paper. Repeat 3× in total.
  f. Wash by picking up one drop of 2% uranyl acetate and then blotting edge on the filter paper. Repeat 3× in total.
  g. Pick up last drop of 2% uranyl acetate and incubate for 5 min.
  h. Remove excess uranyl acetate by blotting.

***Note:*** Uranyl acetate (and filter paper that has been in contact with uranyl acetate) must be disposed of into designated waste.

20. Store grids at room temperature.

***Note:*** Grids should be stored and allowed to dry for at least 30 min before imaging in the microscope**.**

**Pause point:** Stained grids can be kept at room temperature (20°C–25°C) for long-term storage.

21. Collect images using a transmission electron microscope.

A magnification in the range of 11,000× to 15,000 is optimal to obtain images with several filaments in each micrograph.

22. Analysis using Fiji (ImageJ).

Filament lengths can be measured and quantified from the known pixel size.

**CRITICAL:** When comparing the lengths of filaments from different MDA5 mutants/ RNAs, it is important to measure lengths of a sufficient number of filaments from different images/ grids.

### Biolayer interferometry

**Timing: 1 h (per run)**

This biolayer interferometry protocol enables the determination of the affinity and kinetic parameters (association and dissociation constants) for the binding of MDA5 to RNA. The steps of this protocol were performed on an Octet Red384 (ForteBio Inc.) instrument but can also be performed on other Octet BLI systems.

***Note:*** This protocol requires RNA to be biotinylated, for the immobilization of RNA on the streptavidin (SA) Biosensors. Biotinylation of the 3′ end of the RNA molecules is performed prior to BLI experiments using the Pierce RNA 3′ End Biotinylation Kit, according to the manufacturer’s instructions.

**Figure 3:**
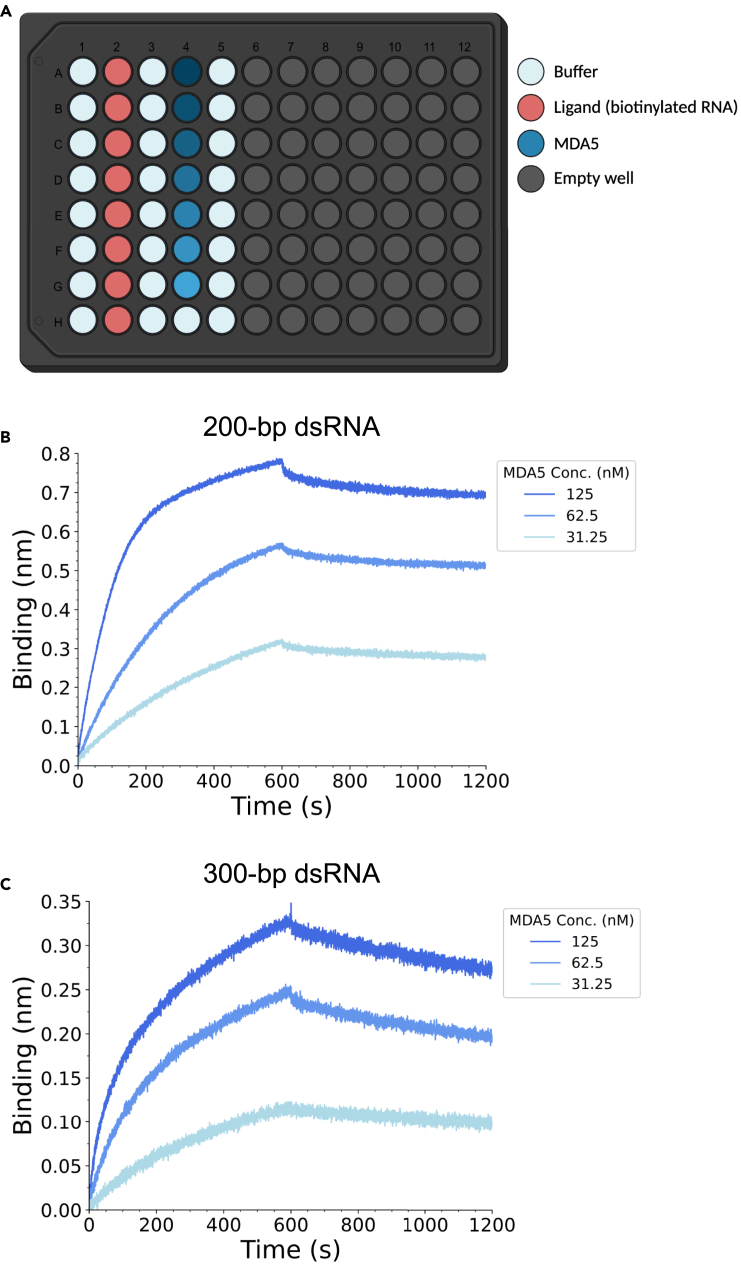
RNA-binding affinities of purified MDA5 using bio-layer interferometry (BLI) (A) Example 96-well plate layout for BLI measurements. Following an initial baseline equilibration step in the assay buffer (column 1), the sensor is loaded with 2.5 ng μL^−1^ biotinylated dsRNA (column 2), followed by a second baseline step (column 3). Association is measured with varying MDA5 concentrations (column 4), followed by dissociation in assay buffer (column 5). For the internal reference sample, assay buffer is used in place of MDA5 for the association step (well 4H). (B and C) BLI curves for the binding of MDA5 to (B) 200-bp or (C) 300-bp dsRNA. 3′-biotinylated dsRNA was immobilized on a streptavidin biosensor, and binding investigated with increasing concentrations of MDA5. Plotted curves are representative data from a single experimental run.

23. Sample preparation.
  a. Prepare dilutions of MDA5 at desired assay concentrations (e.g., 125, 62.5, 31.25 nM) in BLI assay buffer.
    ***Note:*** Additional concentrations within the range of 250–30 nM MDA5 can also be used, and yield data of sufficient quality for kinetic analysis.
  b. Prepare RNA samples, containing 2.5 ng μL^−1^ biotinylated dsRNA in BLI assay buffer.
    ***Note:*** Sample volumes required for each measurement (40–200 μL) depend on the microplate used when running the assay.
    **CRITICAL:** BSA (2 mg mL^−1^) is required in the BLI assay buffer to prevent non-specific binding of MDA5 to the sensors.
24. Create a method file in the Data Acquisition software with the following steps:
  a. Sensor equilibration (baseline): 60 s.
  b. Loading: 600 s.
  c. Baseline: 90 s.
  d. Association: 600 s.
  e. Dissociation: 600 s.
25. Perform the BLI assay.
  a. Transfer samples to individual wells of a black flat bottom microplate, as shown in the Data Acquisition software. See Figure 3A for an example plate layout using a 96-well plate.
    ***Note:*** It is important to run an internal reference sample within each run – for this sample, BLI assay buffer should be used in place of the sample (MDA5) for the association step.
  b. Execute the assay (all steps are performed at 30 °C with shaking at 1000 rpm).
    ***Note:*** Biosensors should be hydrated in BLI assay buffer for at least 10 min prior to all measurements.
26. Data analysis using the Octet Data Analysis HT software.
  a. Select the reference sensor (sample without MDA5) and subtract the assigned reference from all other samples in the run.
  b. In the Data correction tab, align data to the average of the baseline step (align y-axis) and the dissociation step (inter-step correction).
  c. Calculate K_d_ values by fitting the data to a 1:1 binding model (select ‘association and dissociation’ as the step to analyze). In the fitting tab, select the fitting type as ‘local (individual)’ and the fit steps as ‘Full (association and dissociation)’.
  d. Determine average K_d_ and standard error from the K_d_ values obtained from multiple replicates.

## Expected outcomes

This protocol outlines a straightforward 3-step procedure for the purification of MDA5. The protein should be of reasonable purity (>50%) and free from nucleic acid contamination following the anion exchange chromatography step (Figure 1B). In the final SEC step, MDA5 should elute from the SEC column at an elution volume of 13.5–14 mL when using a Superdex 200 Increase 10/300 column (Figure 1D) or around 72 mL for a Superdex 200 16/60 column, corresponding to a monomer. The final yield of the purified protein (>95% purity) after the SEC purification step is typically in the range of 0.6–2 mg/L of *E. coli* culture.

This protocol should yield active MDA5, with both ATP-hydrolysis and RNA-binding activity. Although the measured ATPase activity can vary notably between purification batches, the amount of phosphate generated by following the protocol outlined here should typically be within the range of 10–40 μM using the wild-type mMDA5 protein and 1-kbp dsRNA.^1^ The wild-type mMDA5 has a melting temperature of 44 °C in the buffer conditions described in this protocol (Figure 2A), with a temperature shift of around 10 °C for the corresponding filaments formed upon incubation with 1-kbp dsRNA (Figure 2B). MDA5 will form filaments of varying lengths, within the range of 100–300 nm in length on 1-kbp dsRNA, as visualized using negative stain EM.^1^ Purified MDA5 binds to dsRNA with low nM affinity, as determined by BLI (Figures 3B and 3C), with reported apparent K_D_ values in the range of 1–6 nM for dsRNAs of 200- and 300-bp in length.^1^ This method can be adapted to test the binding of MDA5 to different nucleic acid ligands, although it is worth noting that MDA5 is reported to only recognize dsRNAs longer than 100-bp in length, while significantly longer RNA molecules (1-kbp) result in complex multiphasic binding curves, making them unsuitable for quantitative analysis using the outlined BLI method.^1^

## Quantification and statistical analysis

Melting temperatures were calculated from nanoDSF measurements using the PR.Stability Analysis (NanoTemper) software. Binding affinity and rate constant values from BLI measurements were calculated using the Octet Data Analysis HT software (Sartorius). Prism (GraphPad) was used for statistical analysis of ATPase and BLI data. To quantify filament lengths from negative stain EM micrographs, Fiji (ImageJ) was used.

## Limitations

The *E. coli* expression system described in this protocol allows for the straightforward and fast recombinant expression of MDA5. Due to the nature of the *E. coli* expression system, the expressed proteins may not undergo certain post-translational modifications (PTMs) that would naturally occur during eukaryotic expression. Some variants of MDA5 may exhibit disease phenotypes compared to the wild-type protein due to changes in PTMs that arise as a result of the introduced mutation. In such cases, mutants of MDA5 expressed using this protocol will not display any differences in biochemical activity, relative to the wild-type protein, as the PTM will not be present in either variant. For such variants, an alternative, eukaryotic expression system would be required.

The binding of MDA5 to dsRNA, and subsequent assembly into filamentous structures, is a highly cooperative process comprised of multiple binding events, which can therefore not be accurately quantified by a simple binding model.^5^^,^^7^ While data obtained from BLI measurements for shorter (200- to 300-bp) dsRNA ligands can be fit using a simple 1:1 binding model, this will yield only an ‘apparent K_D_’ for the overall binding event, which does not take into consideration each of the individual binding events. For longer dsRNA ligands (1-kbp), BLI with traces with unconventional multiphasic shapes for MDA5 association are observed, likely due to the formation of RNA secondary structure elements, making this method unsuitable for quantifying MDA5 binding to longer dsRNAs.^1^ However, other methods for quantifying protein interactions, such as isothermal titration calorimetry, would be further hindered by similar issues resulting from the high stoichiometry of the interaction and the dynamic nature of MDA5-dsRNA complexes. Hence, for shorter RNA ligands (100–500-bp), the BLI assay outlined in this protocol provides a suitable method for approximating the kinetic and equilibrium constants for MDA5 binding to RNA, and to compare the binding of different MDA5 variants and RNA ligands.

## Troubleshooting

### Problem 1

Precipitation and changes in the viscosity of the protein sample after thawing frozen aliquots (Step 8).

### Potential solution

Freezing the protein at lower concentrations (less than 1 mg mL^−1^/10 μM), rather than concentrating the pooled fractions from the size exclusion purification step prior to freezing should help with any issues related to increased viscosity. Increasing the final glycerol concentration used for storage from 5% up to 10%–20% can help solve potential issues with protein aggregation and precipitation after thawing of aliquots and may also help reduce decreases in the ATPase activity of MDA5 observed upon freeze-thawing.

The ATPase activity of MDA5 has been observed to increase with increasing concentrations of glycerol present, so caution should be exercised in the case of using higher glycerol concentrations and it is important after thawing aliquots to ensure the complete removal of glycerol (by buffer exchange or desalting), which may otherwise increase variability in subsequent assays.

### Problem 2

There is high background nucleic acid contamination after purification (Step 7).

### Potential solution

Increasing the salt concentration in the initial Ni affinity purification step (Step 5), namely in the lysis and wash buffers, can aid in the removal of nucleic acids bound to MDA5. Increasing the amount of Salt Active Nuclease added to the cell suspension prior to cell lysis can also help with nucleic acid removal. During the column wash step (step 5c), the absorbance should be monitored, and the wash step continued until the absorbance values at both 260 and 280 nm have returned to the baseline level of the wash buffer.

For MDA5 mutants with higher RNA binding affinity, use of a heparin column in place of the anion exchange purification step may help remove nucleic acids from the protein sample, however optimization of purification buffers and the elution protocol would be required.

### Problem 3

There is a high background of free Pi in the ATPase assay buffer, limiting determination of the ATPase activity of purified MDA5 (Step 13).

### Potential solution

The ATPase assay kit used to determine the activity of MDA5 mutants is highly sensitive to the presence of inorganic phosphate (Pi) ions.

As the hydrolysis of ATP into ADP and Pi is spontaneous under the assay conditions, it is critical to run a negative control (Step 10b) to check the ATP stock is not already significantly hydrolyzed to ADP. The ATP stock (4 mM) should ideally be prepared fresh before each run, to ensure integrity of the ATP stock and reduce the Pi background signal.

It is important to also always run protein-only (Step 10b) and buffer-only (Step 10c) controls with every run of the ATPase assay, to check for free phosphate present in protein/RNA samples and assay buffers. In case the background is too high, buffers should be remade and labware rinsed throughly prior to making assay buffers.

Variability in the phosphate generated may also be observed due to the batch and age of the assay kit used.

### Problem 4

No ATPase activity is observed for purified MDA5 (Step 13).

### Potential solution

When testing the ATPase assay of MDA5 variants, it is advisable to also run the wild-type protein in the same assay as a positive control, to confirm whether the lack of activity is due to the specific mutation or issues with the overall assay. The ATPase assay of MDA5 is negatively affected by freeze-thawing or storage at 4°C for multiple days. It is strongly recommended to use freshly purified MDA5 proteins when performing the ATPase assay. The integrity of the RNA stock should also be confirmed (by gel electrophoresis) and heated to > 70°C to remove possible secondary structure elements, prior to the assay. Mg^2+^ is required for the ATPase activity of MDA5, and addition of 4 mM MgSO_4_ in the assay buffer is critical. The presence of inorganic phosphate in assay buffers may also result in lack of perceived ATPase activity, and the ATP stock should be freshly prepared (see troubleshooting, problem 3).

### Problem 5

No filaments are observed in negative stain EM images due to high background and presence of stain crystals on the grids (Step 21).

### Potential solution

A main limiting factor in image quality is the quality of the stain used for imaging. Using a fresh stock of stain and storage at 4°C (avoiding freeze-thaw cycles) can help. Centrifuging the UA stain before aliquoting (Step 19b) should reduce stain crystals on the grids. Additional washing steps with protein buffer after the 5-minute incubation (Step 19g) may also help remove residual dye and stain crystals on the grid. Other stains (such as uranyl formate) can also be used instead of uranyl acetate. In the case that positive staining is observed, increasing the 5-minute incubation step may also help with staining and increasing the contrast of obtained images.

In the case of a high background, it is important to image several areas of the grid, as both staining and protein concentration can vary across the same grid. Dilution of the MDA5-RNA sample immediately before adding to the grid (following the 30-minute incubation, step 18) may also help reduce background signal due to a high protein concentration.

### Problem 6

No binding of MDA5 to dsRNA is observed in the BLI assay (Step 25).

### Potential solution

The signal: noise ratio will be affected by the amount of the RNA immobilized to the sensor during the initial loading step. In the case of insufficient loading of RNA, the loading concentration of RNA or the length of time of the loading step can be increased to allow more RNA to bind to the sensor. Lower immobilization of RNA may also be due to a low efficiency of biotinylation of the RNA. The biotinylation efficiency can be quantified, and troubleshooting performed, by following the manufacturer’s instructions for the Pierce RNA 3′ End Biotinylation Kit. The RNA ligand should also be checked for possible degradation and heated/reannealed to remove possible secondary structure.

For the binding of MDA5, residual RNA contamination from purification can interfere with measured RNA binding and should be addressed as described previously (problem 2). Non-specific binding of MDA5 to the sensor will also affect data quality and can be checked by performing a run with an unloaded reference sensor. It is critical to ensure there are no air bubbles present in the sample wells of the microplate, as this will interfere with measurements.

## Resource availability

### Lead contact

Further information and requests for resources and reagents should be directed to and will be fulfilled by the lead contact, Yorgo Modis (ymodis@mrc-lmb.cam.ac.uk).

### Technical contact

Technical questions on executing this protocol should be directed to and will be answered by the technical contact, Joe Joiner (jjoiner@mrc-lmb.cam.ac.uk) and the lead contact, Yorgo Modis (ymodis@mrc-lmb.cam.ac.uk).

### Materials availability

The plasmid used for mMDA5 expression will be shared by the lead contact, Yorgo Modis (ymodis@mrc-lmb.cam.ac.uk), upon request with a completed materials transfer agreement. This study did not generate any other unique reagents.

### Data and code availability

This study did not generate any new datasets or original code.

## Acknowledgments

We thank Zainab Rashid (VU University Amsterdam) for reviewing and providing valuable feedback on the manuscript. We thank Chris Batters (MRC-LMB Biophysics Facility) and Gail Calvert (Sartorius UK) for assistance with BLI data interpretation. The graphical abstract and Figure 3A were created using Biorender.com. This work was supported by the Wellcome Trust (101908/Z/13/Z to Y.M., 217191/Z/19/Z to Y.M., and 215378/Z/19/Z to R.S.) and the Human Frontier Science Program (LT000454/2021-L to A.H.d.V.). Open-access publication was funded by the University of Cambridge.

## Author contributions

J.D.J.: investigation, methodology, writing – original draft, writing – review and editing, and visualization. A.H.d.V.: investigation, methodology, and writing – review and editing. R.S.: investigation, methodology, and writing – review and editing. Y.M.: supervision, funding acquisition, and writing – review and editing.

## Declaration of interests

The authors declare no competing interests.
